# Past peak prominence: The changing role of integrated assessment modeling in the IPCC

**DOI:** 10.1016/j.isci.2024.111213

**Published:** 2024-10-19

**Authors:** Ema Gusheva, Stefan Pfenninger, Johan Lilliestam

**Affiliations:** 1 Technology, Policy & Management Faculty, Delft University of Technology, Delft, the Netherlands; 2 Sustainability Transition Policy, Friedrich Alexander University Erlangen-Nürnberg, Nürnberg, Germany

**Keywords:** Energy policy, Energy management, Energy modeling

## Abstract

The main task of the Intergovernmental Panel on Climate Change (IPCC) is to provide comprehensive assessments of climate science. However, there are accusations of bias toward certain research fields based on limited empirical evidence. By analyzing the evidence base of Working Group 3 (WG3) reports, we show that integrated assessment modeling (IAM) research was influential in all six assessments, and overrepresented in the Summary for Policymakers (SPM). Further, we show that a small number of men working in Western Europe and the USA dominate IAM research. Thus, global climate negotiations and science may have historically prioritized mitigation solutions suggested by an unrepresentative scientific sample and missed solutions from other perspectives like those of females and non-Western cultures. However, we also show that IAM research influence decreased in AR6, implying a leveling playing field between research fields. But more effort is needed to ensure a comprehensive assessment.

## Highlights

• Empirical proof of epistemic hierarchy in IPCC WG3 with IAM research on top
• IAM research contribution to IPCC WG3 peaked in AR5
• IAM research overrepresented in SPMs by 200% compared to AR
• Limited number of men working in Western Europe and USA dominate IAM research

## Graphical abstract

**Figure:**
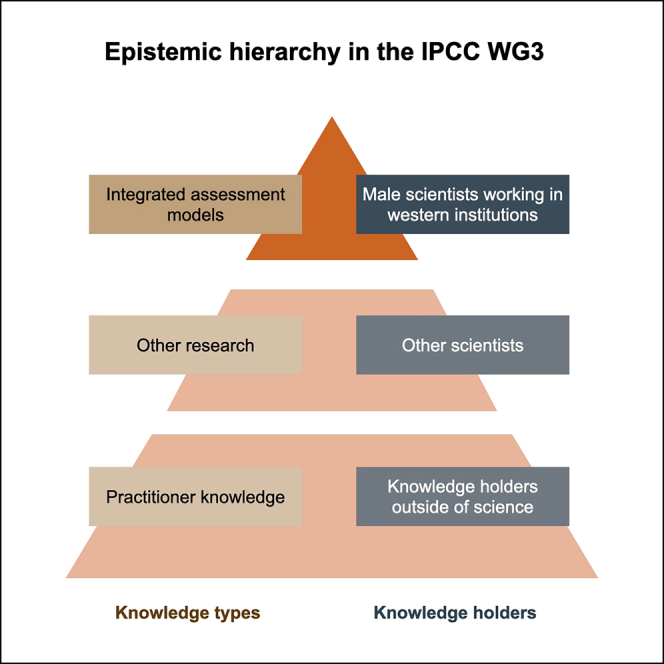

## Introduction

Ensuring a comprehensive assessment of the state of climate change science is the central mandate of the Intergovernmental Panel on Climate Change (IPCC).^1^ Yet, there have been complaints that certain research disciplines have a larger weight in the assessment than others.^2^ This challenges both the legitimacy of the panel and the quality of its findings.^3^ Because the IPCC plays a central role in the science-policy interface, failure to meet the mandate for comprehensiveness has implications for global climate policy and wider scientific culture.

**Figure 1:**
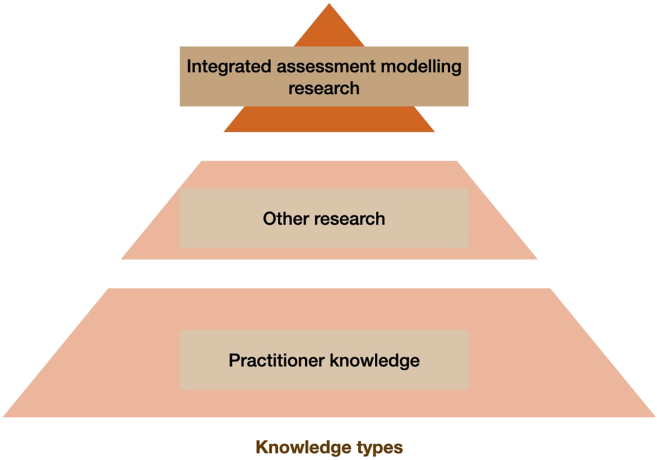
Conceptualization of the epistemic hierarchy in IPCC WG3 assessments The pyramid shows the ranking of knowledge types according to their contribution to the assessment and their resulting power over the story of the ARs. The top and bottom part of the pyramid is based on past research.^4^^,^^5^^,^^6^^,^^7^

The term ‘epistemological hierarchy’ was used to refer to imbalances in knowledge contribution to IPCC assessments;^2^ however, we argue that it is a misnomer since the imbalance does not refer to the philosophical study of knowledge but rather to the way a particular knowledge product (IPCC ARs) is created. Thus, we suggest that epistemic hierarchy is a better term. We use the term ‘epistemic hierarchy’ to provide a theoretical background for exploring the extent to which the IPCC is meeting its mandate for comprehensive assessments. An epistemic hierarchy describes the ranking of knowledge types according to their relative contribution to a knowledge product (Figure 1). In the context of the IPCC, the knowledge product are the scientific assessments presented in the ARs. Hence, an epistemic hierarchy would be observable if certain types of knowledge play a stronger role than others. In the constructivist view, knowledge is tied to and should be understood in relation to the people that produced it. It follows then that the existence of an epistemic hierarchy also posits that certain people play a stronger role in the assessment compared to other people.

Past research has identified asymmetries in the treatment of knowledge in WG2 and WG1. For example, in the WG2 report for AR5, there was limited inclusion of indigenous content in the chapter on human health despite there being rich literature on it, failing to recognize that the vulnerabilities of indigenous health are unique and place-specific.^4^^,^^8^^,^^9^ Similarly, within WG1, the results from process-based models were valued more than estimates from paleoclimatology, which led to the exclusion (in AR4) and underestimation (in earlier reports) of estimates on sea-level rise, to the detriment of both policymakers and the decisions made in response to the published statements.^10^ Past work also showed that the authorship of WG3 was dominated by scientists from only a few research institutions.^11^^,^^12^^,^^13^^,^^14^ Although the IPCC has long held guidelines for its author selection process to specifically seek diversity in gender, country of residence, and academic track record of authors,^15^ it is only recently that the gender and geographical distribution of authors has improved.^16^^,^^17^ However, an improvement in the diversity of authors does not directly translate into an improvement in the diversity of evidence assessed. There is little empirical research on the evidence base of the IPCC reports, and it is limited in scope. The evidence base of AR3 lacked political, ethical, and cultural analyses^18^^,^^19^ which inspired calls for more inclusion of anthropology,^20^ wider social science,^21^^,^^22^ humanities,^23^^,^^24^ practitioner knowledge,^5^^,^^25^ technical solutions from engineering, technology and agricultural sciences^26^ and emerging scientific findings which do not yet have wide support.^27^ Despite such findings and calls for more diversity, the suspicion of an epistemic hierarchy challenging the comprehensiveness of the reports has not been supported – or rejected – by empirical research.

Integrated assessment modeling (IAM) research is an interdisciplinary research field that is nonetheless an epistemologically homogeneous group with a shared approach based on representation in computer models^28^ and a paradigm that views climate change mitigation as an economic problem.^29^ As such, it is intimately linked with the IPCC.^6^^,^^7^^,^^30^^,^^31^ IAMs have been included since the first report,^28^ but starting with AR4, the management of this research was outsourced to the Integrated Assessment Modeling Consortium (IAMC).^32^ As it is based on templates developed by the IAMC, the scenario database for AR6 has been criticized for favoring results from IAMs, impeding submission of results based on models with different reporting variables than IAMs and leading to a non-representative database of scenarios.^33^^,^^34^^,^^35^ Simultaneously, IAMs are subject to increasing criticism such as their fitness-for-purpose,^36^ failure to account for ethical considerations,^37^ lack of transparency^32^ and other methodological shortcomings.^38^^,^^39^ The role of IAMs in the IPCC reports is estimated to have grown over time,^7^ but there is no detailed empirical evidence, and especially no quantitative evidence. Here, we investigate the prominence (or lack of it) of IAM research in the evidence base for the WG3 assessment reports and Summaries for Policymakers, and explore whether an epistemic hierarchy exists, with IAM research at the top of it.

The existence of an epistemic hierarchy risks undermining the credibility of the IPCC in the eyes of those excluded from the assessment.^40^ It also risks missing some solutions altogether by overemphasizing mitigation solutions propagated by one disciplinary perspective; specifically in the case of IAM research, it could lead to overly relying on insights based on an economic paradigm based on critical non-explicit uncertain assumptions about future global socioeconomic and sociotechnical developments. Further, an epistemic hierarchy affects broader scientific culture given the impact the IPCC has on scientific output.^41^ The privileged status of a single discipline or research field would mean that more funding would be made available for it, likely to the detriment of others.^10^^,^^42^ In the case of IAM research, it may contribute to the prioritization of quantitative research over qualitative research,^43^^,^^44^ which, in turn, can reinforce a non-inclusive scientific knowledge culture biased toward a singular definition of good and useful research.

Our study is a contribution to the reflexive turn in science,^45^^,^^46^ wherein the research focus has shifted inwards, toward the researchers themselves. We address the question of the existence of an epistemic hierarchy in scientific assessments like the IPCC and discuss the consequences for both science and policy. Given that models act as boundary objects in the science-policy interface, our work contributes to recent studies finding evidence of political calibration of models^30^^,^^47^^,^^48^ – policy-based evidence-making as opposed to the ideal-typical model-based policymaking.^49^ By studying the case of the role of IAM research in IPCC ARs, we offer robust and quantitative empirical data for discussing the effect of models on policy and vice versa.

We apply semi-automated scientometric reference analysis to all 33,585 references from the six WG3 assessment reports. To quantify the IAM research evidence base, we first measure what proportion of all references is authored by IAM researchers and how many of the most cited researchers are IAM researchers. We operationalize the concept of “IAM research” through a bottom-up approach, by looking at the most cited researchers per report, and a top-down approach, by listing the principal researchers of the IAMs who have contributed the most to the IPCC. Second, we analyze the citations of all 2,108 factual statements from the six Summaries for Policymakers (SPMs) and match them to the evidence base of the chapters they cite. We test the sensitivity of our results to different IAM research operationalizations and evidence base benchmark levels (see Experimental Procedures for the full method description).

Our study offers three findings. First, we show that IAM research is overrepresented in the SPMs compared to its contribution to the ARs. This implies that IAM research findings are given a strong voice in the most-impactful text of the report, possibly at the expense of findings from other research fields. Second, in comparison to other studies on the role of IAM research that do not include AR6, we show that its role decreased in the latest assessment cycle. Our research builds on existing studies based on qualitative research methods that have suggested that IAM research is central to IPCC assessments,^6^^,^^7^^,^^48^ but is unique in analyzing data from AR6. By including it in the scope of our analysis, our results show that the prevalence of IAM research has shrunk. Third, we show that IAM research is conducted by a small and highly homogeneous group of mostly male researchers residing in Western institutions. Such a lack of diversity highlights issues of potential bias in IAM research output and warrants serious investigation given its prominent role in the assessments.

## Results

### IAM research was overrepresented in the SPMs, but it fell in prevalence in AR6

**Figure 2:**
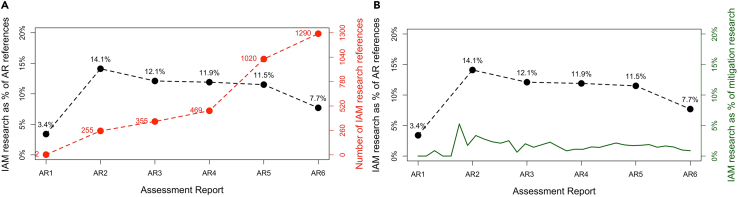
Relative size of IAM research field within climate change mitigation science and its contribution to WG3 reports (A) Shows the distribution of IAM research across all WG3 reports as a share of all reference (left axis) and in absolute numbers (right axis). (B) Compares the contribution of IAM research to the reports (left axis) with its contribution to all scholarly literature on climate change mitigation (right axis).

IAM research is prevalent in WG3 reports: on average, 10.2% of all references across the assessment reports were IAM research (Figure 2A). IAM research has been estimated to form 0.5–1% of all climate change scholarly literature in the period from 1989-2019.^7^ Our analysis confirms this and further shows that IAM research forms a much larger part of the evidence in WG3 ARs compared to its relative size within climate change mitigation literature – between three and eight times larger (Figure 2B). Hence, IAM research is overrepresented in the IPCC ARs compared to its contribution to climate change mitigation research, which suggests that its prevalence in IPCC ARs comes at the expense of other research fields.

IAM research was a strong contributor to all six ARs, but other research fields have gained a stronger role in evidence provision. As a share of all references, IAM research shows a slightly falling trend since AR2, with the strongest drop in AR6 (Figure 2A). Although AR4 and AR5 have similar IAM reference shares, the absolute number of references more than doubled between the reports. The absolute number of references increased again between AR5 and AR6, but the relative share of IAM research decreased. This suggests that the contribution of other research fields grew at a faster rate than that of IAM research. The relative size of the IAM research field has started to decrease since AR5 (Figure 2B), providing a possible explanation for why the prominence of IAM research has decreased in AR6.

**Figure 3:**
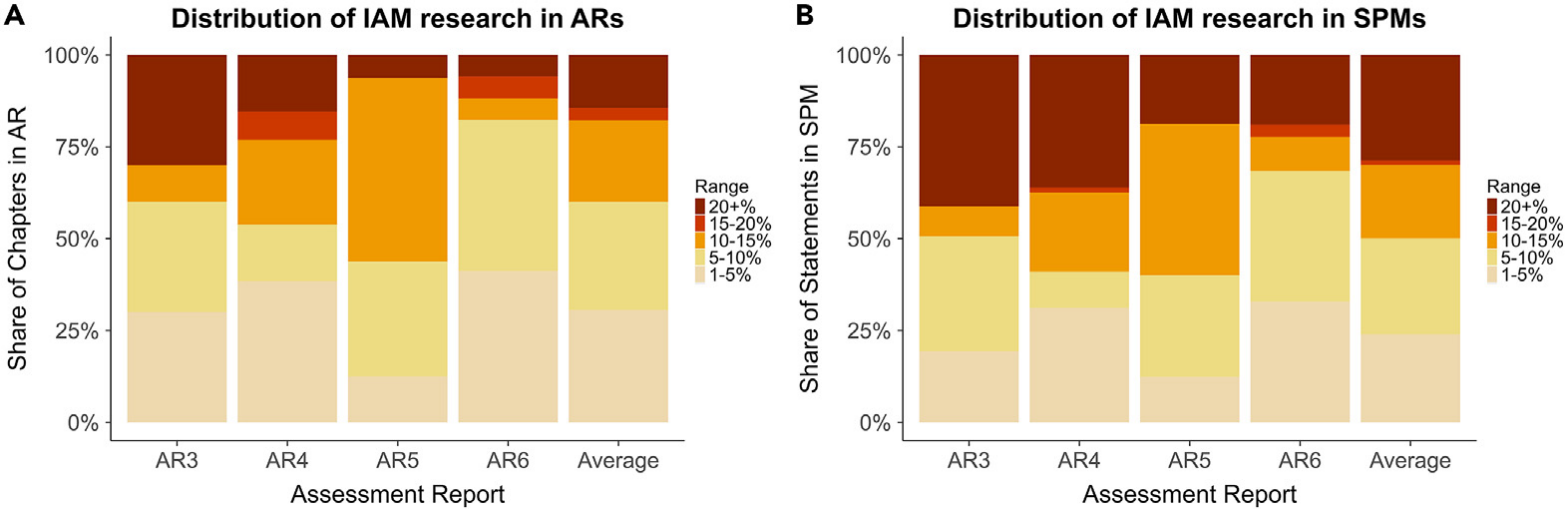
Distribution of different IAM research reference shares in the full report and in the SPM per assessment cycle (AR3-AR6) (A) Shows the portion of all chapters that had a given IAM research reference share, presupposing that all chapters are of equal importance for the full report. (B) Shows the portion of all SPM citations to a chapter with a given IAM research reference share. Results for AR1 and AR2 are excluded because the SPMs of these reports do not include citations. See Figure S3 for an extended version of 3a that includes AR1 and AR2.

Evidence from IAM research was distributed across all chapters (Figure 3A): every chapter in AR3-AR6 had an IAM reference share exceeding 1% of references, showing that all parts of the reports contain IAM research to some extent. Further, IAM research was prevalent (exceeding 10% of references) in almost half of all chapters (45%) and highly prevalent (exceeding 20% of references) in 13% of all chapters (see Figure S3 for chapter-level results from AR1 and AR2). However, in AR6, the distribution of IAM research was largely contained in just a few chapters and the remaining chapters were dedicated to issues not or not strongly informed by IAM research. For example, although IAM research was prevalent (>10% of all references) in over 60% of AR5 chapters, IAM research was prevalent (>10%) in only 20% of chapters in AR6. This highlights that IAM research played a weaker role in AR6, and not only that the report structure was different from earlier reports.

We find that IAM research is overrepresented in the SPMs compared to its share of evidence in the reports. Chapters where IAM is highly prevalent (>20% of all references) form a larger portion of all SPM citations (Figure 3B) compared to their contribution to the overall report (Figure 3A). For example, although IAM research is highly prevalent (>20% of all references) in only one chapter in AR5 and AR6 (6% of all chapters), 19% of all SPM citations are to this one chapter and more than 30% of all SPM statements cite it (Figure S4). This suggests that, in the SPM, IAM research is often co-cited with knowledge from other fields. Overall, chapters where IAM research is highly prevalent (>20% of all references) contributed to on average 14.4% of the full report but to 28.8% of the SPM in AR3-AR6. This shows that IAM research are overrepresented in the SPM compared to its share in the ARs (Figure 3): in the SPMs, which are the most policy-relevant output of the IPCC, IAM prevalence is double that of the reports.

### IAM research was dominated by a dozen mostly male researchers working in a handful of Western countries

**Figure 4:**
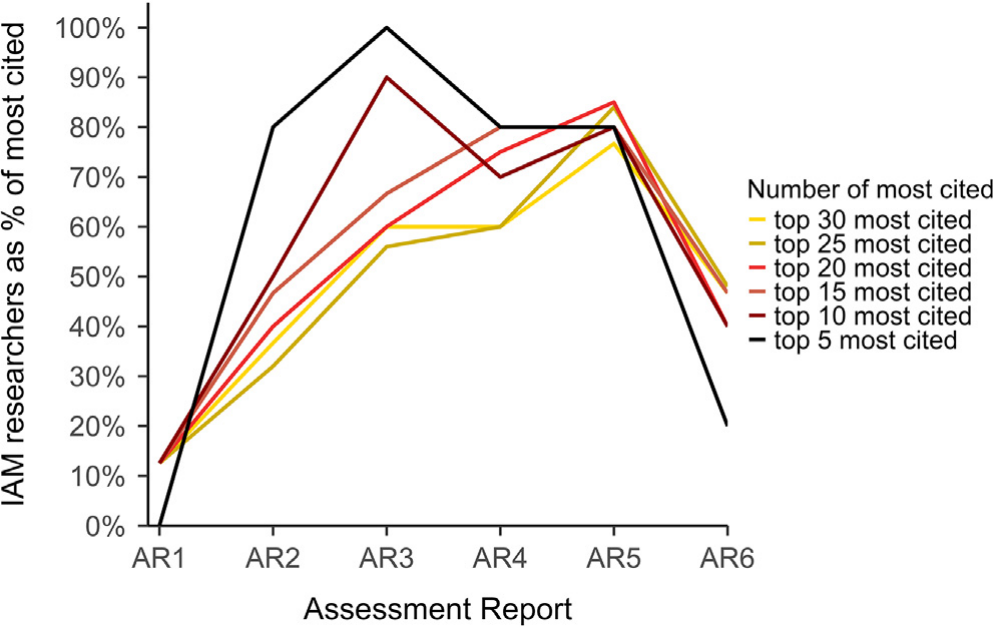
Share of IAM researchers in top 5–30 most cited researchers per report (AR1-AR6)

A small number of IAM researchers are dominant, both across all research that contributed to the reports and IAM research specifically. IAM researchers had a high incidence in the list of the most cited researchers per report (Figure 4), with all five most cited researchers in AR3 being IAM researchers and 70% of the top 30 most cited researchers in AR5. In all reports since AR3, IAM researchers were 45% or more of the top 30 most cited researchers. This shows that IAM researchers were dominant (∼50% of 30 most cited) contributors to the evidence base of the reports, although their importance decreased somewhat in AR6.

**Figure 5:**
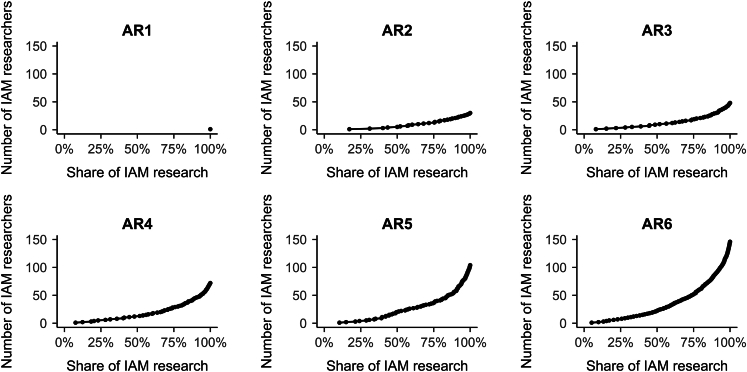
Attribution of IAM research to individual researchers, per report (AR1-AR6) The x axis represents the number of IAM researchers, while the y axis represents the share of IAM research in the report. Each dot represents how many researchers have (co-)authored a given share of IAM research in the full report.

The contribution of individual IAM researchers to IAM research in the report is skewed in favor of a few individual researchers (Figure 5). On average, just twelve researchers contributed to more than 50% of IAM research per report (see Table S1 for list). In AR1, a single IAM researcher (co-)authored all IAM references; and five researchers were authors of more than 50% of all IAM references in AR2. In AR3, more than 50% of the IAM references were first- or co-authored by just ten researchers, in AR4 by 13 researchers, and in AR5 and AR6 by 21 and 23 researchers, respectively. This shows that a very limited number of individuals dominate IAM research covered in the reports, although there is a trend toward somewhat more diversity in IAM research over time. For AR2-AR6, the majority of the dominant IAM, i.e., researchers who together (co-)authored more than 50% of IAM research, were also authors of the AR (Table 1). That may explain why IAM research was so prevalent in the ARs, but it does not explain the change in IAM research prevalence for AR6 (Figure 2A) because a larger share of IAM researchers are also AR authors in AR6 compared to AR5, yet the relative prevalence of IAM research decreased.

**Table 1:** AR authorship of dominant IAM researchers

AR	Share dominant IAM researchers who also authored the AR
AR1	0%
AR2	60%
AR3	100%
AR4	92.3%
AR5	76.2%
AR6	82.6%

The table shows how many of the dominant IAM researchers who together (co-)author more than 50% of IAM research in each report (as shown in Figure 5) were AR authors. See Table S1 for the names of the researchers.

**Figure 6:**
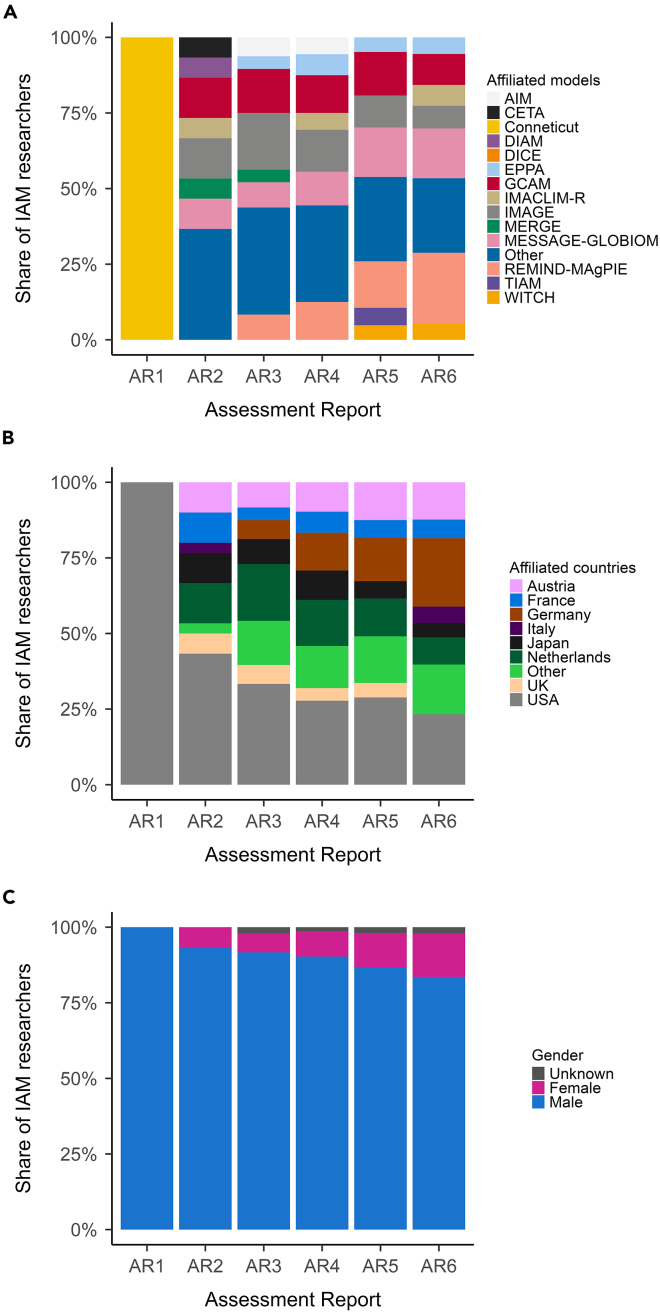
Change in the diversity of IAM researchers across reports Distribution of affiliated models (A), countries (B), and gender (C) for all IAM researchers who have been cited in WG3 reports.

The group of researchers who contributed to IAM research in the reports is highly homogeneous. Most IAM researchers are affiliated with one of four models, consistently across all reports (Figure 6A): GCAM, IMAGE, MESSAGE, and REMIND. Consequently, most IAM researchers work in a limited number of countries (Figure 6B), particularly the USA, Germany and the Netherlands (>50% of all IAM researchers in our sample across all reports) with the largest number of researchers affiliated with institutes in the USA. By far most IAM researchers are male, with a slight improvement in gender distribution over time (Figure 6C). Thus, the results show not only that a limited number of IAM researchers are dominant (listed in Table S1), but also that the IAM research community lacks diversity overall.

## Discussion

There are fears of an epistemic hierarchy in IPCC assessment reports, with suggestions that IAM research is at the top of that hierarchy for WG3, implying bias in the assessment. Our results show that IAM research was prevalent in all IPCC assessment cycles, overrepresented in the SPMs and that it was predominantly provided by a highly non-diverse group of male researchers working in Western institutions. We thus conclude that there is an epistemic hierarchy in WG3 assessments, overemphasizing some perspectives while underestimating others. And, although we do not investigate whether another research field could be even higher up, IAM research is likely at the top of that hierarchy, suggesting that it is influential in shaping the story of the ARs. However, we also show that the prevalence of IAM research shrunk in AR6, implying that its influence may have peaked and that suggests that the hierarchy may have already started to flatten.

Our results imply that IAM research was influential in WG3 assessments. Thus, it has had a larger weight in shaping the narrative of the ARs compared to other research fields, possibly crowding out alternative narratives. Previous research suggests that this may be because no other research field has achieved a comparable degree of systematic synthesis of research in a policy-relevant manner.^22^^,^^50^^,^^51^ Thus, IAM research may be influential because of the shortcomings of other disciplines. For example, it may have come to substitute ex-post empirical policy evaluation studies in earlier reports,^52^ although, being an *ex ante* tool, it is unsuited for this substitution.^53^ Alternatively, the influence of IAM research in the IPCC may be explained by the selection of the IPCC Bureau^54^ and report authors as the share of Bureau members and report authors who are IAM researchers or their close collaborators can affect the prevalence of IAM research in the reports. Our results hint at this as we show that the majority of dominant IAM researchers in AR2-AR6 were also AR authors. Similarly, previous research has found that engineers and economists, which is the usual disciplinary background description of IAM researchers, dominate WG3 AR authorship.^11^^,^^13^

We show that IAM research is overrepresented in the SPMs, with IAM-research-heavy chapters supporting more than twice the share of statements in the SPM compared to the role they have in the corresponding reports. In other words, the SPMs amplify statements emanating from IAM research at the expense of statements from other fields. This result may be explained by IAM-heavy chapters appearing earlier in the reports compared to other chapters. When compared to later chapters, earlier chapters receive more negotiation time in the SPM approval process,^55^ increasing the likelihood of reaching a compromise text and decreasing the likelihood of having the text deleted. Alternatively, the result may be explained by it being easier to compromise during negotiation on this text than text supported from other research fields. Previous research has argued that IAM research is global and decontextualized,^56^ and, thus, does not implicate any specific country or action,^22^ or that IAM research findings are not immediately actionable, not raising the pressure for policy reforms. If so, that suggest that political actors in the IPCC, specifically representatives of IPCC member countries in charge of approving the SPM, are more sympathetic to IAM research findings as opposed to findings from other types of research, possibly because it does not commit them to any specific action. However, IPCC procedures allow for contentious issues on parts of the text to be covered in footnotes, which incentivizes compromise seeking and disincentivizes text elimination.^57^ Lastly, given that the IPCC is a global (not local) organization, the result can be explained with the argument that SPMs are more interested in findings from global-scale research like IAM research compared to the full report where there is more space to discuss findings from more local-scale research.^7^^,^^58^

Not only was IAM research influential within the IPCC, but IAM research itself was strongly dominated by a handful of men from Western institutions. We show that of the 30 most cited researchers in the WG3 reports, around 50% were IAM researchers in all reports since AR3, further strengthening our finding of IAM research influence in the IPCC. Within the influential position of IAM research, just twelve researchers (co-)authored the majority of IAM research on average per report. Because the number of researchers responsible for most of the contribution is so small, it is also accompanied by strong geographical and institutional skewness, with almost all IAM researchers residing in the US and Western Europe, almost all of which were male, and affiliated with a handful of models. This further emphasizes our finding of an epistemic hierarchy, prioritizing only one specific type of knowledge.

The role of IAM research in IPCC WG3 seems to have peaked. Our results show that the share of IAM research out of all references decreased in AR6 even though the absolute number of IAM references increased, suggesting that the contribution of IAM research grew, but not as fast as that of other research fields, negatively affecting its overall relative influence. This may be a direct effect of the Expert Meeting recommendation to include critics of IAM research in the author list.^32^ Another reason may be that the role of IAM research was diluted because the number of chapters grew over time, explicitly generating room for new types of insight, especially action-oriented insight, which differs from the target- and cost-centered key output of IAM research. However, we find that a more likely explanation is that the relative size of IAM research within climate change mitigation research has decreased. Thus, although our results suggest that the IPCC has managed to increase the diversity of disciplines, perspectives and backgrounds and has progressed toward reaching its mandate of ensuring comprehensiveness, it may simply be a reflection of a change in the scientific community and literature rather than a result of something the IPCC has done.

Our findings are in line with existing research on the role of IAM research in IPCC reports. Previous research on the Synthesis Reports (we analyze the WG3 Assessment Reports) found that the contribution of IAM research steadily increased reaching the highest value in the AR5 cycle.^7^ Our results agree with the trend until AR4, but while we show that the absolute number of IAM references and the share of top-cited IAM researchers grew in AR5, IAM research did not increase its share of the evidence, neither in the report nor in the SPM. Our findings are likely different because they are more detailed and applied to a different set of documents. However, both our and past studies agree that IAM research plays an influential role in IPCC assessments.

Two important implications stem from our findings. First, the influence of IAM research in WG3 may have (and continue to) adversely impacted science and policy by pushing it to focus strongly on mitigation ideas proposed by IAM research and overlook other ideas more strongly supported by other research fields. For example, given the overrepresentation of IAM research in the SPMs and the SPMs’ instrumental role in UNFCCC negotiations,^56^^,^^59^ it may have pushed the prioritization of BECCS^60^ over other technical or behavioral mitigation solutions. It may have also limited the usefulness of the IPCC for policymaking because of the prioritization of a global decontextualized narrative^58^ over solution-oriented and context-specific content in the reports^26^^,^^52^^,^^61^; the inclusion of carbon pricing as the only explicit policy instrument in IAMs likely contributed to framing carbon pricing as a must-have, despite its weak empirical track record.^53^^,^^62^ Further, our finding that IAM research is dominated by males working in Western institutions implies that potentially strong biases may have (and continue to be) spilled over to science and policy whereby the scientific and policy consensus for all is defined by a few individuals with highly similar backgrounds, consequently placing those from other affiliations at an advantage. It creates a situation in which science, science assessment and policymaking are done predominately by men from developed countries despite women and from developing country citizens being more vulnerable to climate change.^63^^,^^64^

Second, we find evidence that the relative influence of IAM research is decreasing for the IPCC, but there may be negative implications from a flattening epistemic hierarchy. Our finding that IAM research is spread across all chapters of the reports and a disproportionate share of SPM statements suggests not only an IAM research overrepresentation but also that IAM research plays a knowledge-bridging function between chapters.^14^ This is because the results show that IAM research is considered side-by-side with all knowledge in the ARs by all chapter authors and especially SPM authors. Thus, if the role of IAM research were to decline further and no other fields were to shoulder this knowledge-bridging function, it may reduce knowledge transfer between chapters. This is important both for ensuring the coherence of the main messages from the report, but also for inter- and transdisciplinary deliberation for future scientific advancement. However, future research applying network analysis can shed more light on the specifics of this knowledge-bridging function as well as reveal whether the decreasing role of IAM research implies a risk for polarization in the assessment.

We thus call for the IPCC Bureau and the IAM research community to apply serious efforts to increase the diversity of climate change mitigation knowledge providers and consequently flatten the epistemic hierarchy in IPCC assessments. Both the IPCC Bureau and the IAM research community must advocate for inclusive knowledge cultures because of the central position they play in the science-policy interface. IAMs are global models and thus, normatively speaking, they should represent all perspectives and values evenly. But that is difficult to do when the modelers are mainly located in Western institutions. Encouraging IAM development in institutions in non-represented countries and institutions and promoting gender equality in IAM teams can go a long way in increasing the legitimacy of IAM research. Alternatively, IAM research can benefit from more serious efforts to compare their results to similar results from non-Western institutions. On the other hand, the IPCC Bureau can contribute toward an inclusive knowledge culture by actively encouraging the assessment of research fields underrepresented in the ARs,^26^ especially in its author meetings. Further, it can highlight the IPCC’s aim for comprehensiveness in attempts to strive for a fairer representation of AR findings in SPM approval meetings. This would help avoid the overrepresentation of certain research fields, like IAM research, in the SPMs.

Previous research has highlighted the knowledge hierarchy in WG3 assessments, suggesting that scientific knowledge is valued more highly than interpretative or indigenous knowledge. We demonstrate that this hierarchy also exists within scientific knowledge and that IAM research and a handful of IAM researchers occupy a high, possibly the highest, position in it. This knowledge contribution asymmetry, which we conceptualize as an epistemic hierarchy, is at odds with the IPCC’s aim for a comprehensive assessment and has implications both for science and policy. It contributes toward a scientific culture in which IAM research is reinforced, while other types of research receive less material conditions for progress; and toward a policymaking environment that prioritizes solutions from a single research field while missing solutions from other fields. Further, the finding that IAM research is dominated by male scientists residing in a handful of Western countries implies that there may be biases that spill over to science and policy. But our results also imply that IAM research has reached peak prominence in AR5, suggesting that the epistemic hierarchy in WG3 assessments may have started to flatten. The trend is encouraging, although much remains to be done to promote diversity in the evidence base of IPCC assessments.

### Limitations of the study

Our article provides empirical evidence for the presence of an epistemic hierarchy in WG3 assessments, but it also has some limitations and uncertainties. We show that an average of 10.2% of all the evidence across all the reports was IAM research and interpret this as “prevalent” because research on the size of the IAM research field has shown that the relative share of IAM publications in academic climate research ranges between 0.5% and 1%^7^ and we show that it ranges between 1% and 5% within climate change mitigation research specifically. However, further research needs to show whether other research fields have contributed to a comparable extent to determine to what extent 10% can be considered only “prevalent”, “highly prevalent” or even “dominant”. Similarly, further work is needed to discuss what is an appropriate contribution of different research fields, given that not all literature is equally policy-relevant, which is another one of IPCC’s mandates.^26^

We do not claim that our results are quantitatively precise. Our results for earlier reports, especially AR1, may be an underestimation since IAM research was done internally as part of the IPCC process, so it may not have been referenced. We also assume that all the publications of researchers operationalized as IAM researchers (see details on how we operationalize IAM research in experimental procedures) are, in fact, IAM research. This leads to an overestimation of our results, because researchers are classified as an IAM researcher if they have (co-)authored at least one cited article applying an IAM. However, this uncertainty is unlikely to qualitatively affect our conclusions because the results have the same trend for all sample sizes above the 15 most prominent IAM researchers (Figure S2).

## Resource availability

### Lead contact

Further information and requests for resources should be directed to and will be fulfilled by the lead contact, E.G. (e.gusheva@tudelft.nl).

### Materials availability

This study did not generate new unique materials.

### Data and code availability

• The data generated during this study have been deposited at 4TU.ResearchData and is publicly available under the accession number listed in the key resources table.
• The code for analysis has also been deposited at 4TU.ResearchData and is publicly available under the accession number listed in the key resources table.
• Any additional information required to reanalyze the data reported in this paper is available from the lead contact upon request.

## Acknowledgments

This research is a part of the lead author’s doctoral dissertation, which was funded by the 10.13039/501100000780European Commission as part of the European Climate and Energy Modeling Forum. Grant agreement ID: 101022622.

## Author contributions

E.G.: Conceptualization, Methodology, Investigation, Writing – Original Draft, Visualization. S.P.: Conceptualization, Writing – Review and Editing. J.L.: Conceptualization, Writing – Review and Editing.

## Declaration of interests

The authors declare no competing interests.

## STAR★Methods

### Key resources table

**Table:** 

REAGENT or RESOURCE	SOURCE	IDENTIFIER
**Deposited data**		

WG3 assessment reports	IPCC	https://www.ipcc.ch/reports/
Processed references and data, text mining scripts and result data	This paper	https://doi.org/10.4121/ec2fffe3-0bd7-4a7b-b477-74b25ad17983

**Software and algorithms**		

R Studio	Posit PBC	posit.co/products/open-source/rstudio

### Method details

#### Method overview

We use scientometric reference analysis to calculate the share of IPCC WG3 AR references that comes from IAM research (Figure S1). We first operationalize IAM research by identifying prominent IAM researchers. Second, we calculate the share of IPCC report references authored by IAM researchers using semi-automated scientometric scripts. Third, we analyze the citations of all SPM statements and match them to the evidence base measured on a chapter level. Last, we analyze the model, gender and geographical location of IAM researchers’ affiliated institutions in combination with their contribution to the ARs.

#### Dataset

We review all six WG3 IPCC assessment reports. Within it, our focus is on the list of references at the end of all chapters and the content of the SPMs. In total, we analyzed 143 pages of 2108 sentences in the SPMs and 33,585 references distributed across 6 reports and 71 chapters. Within the SPMs, we analyzed 1312 citations across 1362 statements. To prepare our data we machine-read the references and calculated how many references there are per report and chapter as well as manually counted how many citations there are per SPM.

#### IAM research operationalization

We used a bottom-up approach, which identifies IAM researchers by looking at the most cited researchers per report, and a top-down approach, which identifies IAM researchers by identifying IAMs and their principal researchers. Thirty-four IAM researchers were found by both approaches. We filtered out researchers who had no contribution to any of the reports. We then combined the results into a final cumulative list of 185 IAM researchers, 127 based on the top-down approach, 23 based on the bottom-up approach, and 35 which were identified with both approaches. The list is representative of IAM research spanning the period from 1990 to 2022. It includes researchers who were prominent in the 1990s but have since retired, as well as younger researchers who did not appear in early reports but are prominent in the latest reports. This way, we can be certain that we did not overestimate the number of IAM researchers, but that, if anything, our list of researchers is incomplete, thus it underestimates the role of IAM research.

#### Bottom-up approach for identifying IAM researchers

We machine-read the references of all chapters and integrated them into a single list per report. Next, we cleaned the data to contain solely the author’s names and omitted other parts of the references. This way, our results exclude author names appearing in a later part of a reference, such as book editors or authors mentioned in the title of a publication as is the case in rebuttals, commentaries or other response papers.

Using a text-mining technique for identifying the most common words in a text document based on a Document-Term Matrix (DTM), we listed the most commonly occurring last names in the list of references. We opted for the approach of searching the most common words because it is reliable and computationally less intensive than text comparison approaches like cosine similarity or partial string matching. After identifying the most common words, we cleaned the data by filtering out instances of the reports citing other IPCC reports, global development institutes, banks, or governments as well as to control for instances where multiple researchers share the last name. The output of this was a list of the most cited surnames in each report. After controlling for instances of surnames shared by more researchers, we coded researchers in our list as either “IAM” or “non-IAM”. This decision was based on whether the researcher has authored at least one peer-reviewed paper applying an integrated assessment modeling method. Co-authorship or a single IAM-based paper may not be enough to characterize a researcher as an “IAM researcher” and one may argue against classifying certain researchers as IAM researchers. To control the impact of this, we performed sensitivity testing to see how changing the sample of IAM researchers affects our results and found that it doesn’t qualitatively change the results beyond the 15 most prominent IAM researchers and the quantitative change beyond the 95 most prominent researchers is negligible (Figure S2). For the bottom-up approach, we started by identifying the 5 most cited researchers and measuring what portion of them were IAM researchers. Next, we kept increasing the size of our sample by 5 until we reached saturation whereby the effect of the number of most cited researchers considered on the resulting share of IAM researchers stabilized (see Figure 3 in Results to see that the trend was stable starting from the 15 most cited). The bottom-up approach resulted in a list of 58 IAM researchers, 35 of which were also found by the top-down approach.

#### Top-down approach for identifying IAM researchers

We made a list of the most published IAMs by looking at which IAMs had appeared both in AR2^65^ and AR6^66^ and those that had most commonly participated in model intercomparison projects. We developed a list of the IAMs by looking at the IAMs listed in the chapter on review of IAMs in AR2, the models that have been most commonly part of MIPs, and those who contributed a great deal of the scenarios to AR6 (Table S2).^6^^,^^67^^,^^68^ Next, we identified principal researchers associated with each model on the list by emailing a prominent researcher for each model and requesting they list the principal researchers in charge of the model between 1990 and 2022. We took the names listed as principal modelers in AR2, the IAMC or the names of authors of model documentation for the models for which we could not get an email response. Due to this mixed data collection approach, we list principal researchers for the models for which we could get primary information via email correspondence. Our list of associated researchers is more extensive, and includes junior researchers, for the models for which we consulted model documentation. Including only principal researchers may have led to an underestimation of our results. Whereas, including associate researchers may have led to an overestimation since the chances are higher that an associate researcher has done non-IAM research. We filtered out the names of researchers which have not been cited in any of the IPCC reports. In the end, this approach yielded a list of 20 models and 162 researchers, 35 of which were also found by the bottom-up approach.

### Quantification and statistical analysis

#### Analysis of IAM research contribution to ARs

We calculated the share of references authored by all IAM researchers both separately per AR and together, considering that some of these researchers have co-authored publications. Specifically, we used the “tm” package in R to process the text in the reference lists and split it into words. Next, we used string matching to identify all references that contain the surname of an IAM researcher and calculated their number. We manually reviewed all results and corrected the results for researchers without unique surnames to ensure that the reference did not cite another researcher with the same surname.

To understand how widely spread the contribution of IAM research is within the reports, we analyzed the results on a chapter level (each chapter has its own reference list and the sum of all the chapter reference lists is the total reference list of the report). We chose different baseline levels for IAM research contribution (0–1%, 1–5%, 5–10%, 10–15% and 20%+ of the references) and calculated the spread across chapters. We tested the sensitivity of our results to the sample size of IAM researchers included in the analysis. After ranking the list of IAM researchers based on the magnitude of their impact, we varied the sample of researchers from 185 (full sample from both approaches) to 5 in steps of 10. The overall trend is the same across different sample sizes of IAM researchers starting with the 15 most prominent researchers (Figure S2). This supports the argument that the research of the 15 most prominent IAM researchers is enough to explain the overall pattern describing the role of IAM research in the IPCC reports. Increasing the sample size of IAM researchers above the 105 most prominent IAM researchers gives arbitrary increases in the resulting share of references. This means that the research of just 105 researchers is enough to derive results close to our final results, which were based on 185 researchers. (Figure S2).

We benchmarked our result on IAM research contribution to ARs with a quantification of the relative size of the IAM research field within climate change mitigation scholarly literature. Under the assumption that the IPCC aims for perfect representation of science, this benchmarking investigates whether IAM research is over or under-represented in the ARs. We based our quantification on a related study that estimated the size of IAM research within climate change literature more broadly.^7^ Specifically, we ran a Scopus search using the query ("climate change" OR "greenhouse gas" OR "global warming") AND ("mitigation" OR "abatement" OR "reduction") to identify academic climate change mitigation literature and the query ("climate-economy model" OR "integrated assessment model" OR "integrated assessment modeling" OR "integrated assessment modeling" AND "climate") to identify academic integrated assessment modeling literature (same as previous research^7^) within publication titles, keywords and abstract text.

#### Analysis of IAM research contribution to SPMs

We analyzed all citations in all SPM statements AR3-AR6 and calculated their evidence base as it relates to different chapters. We did not consider AR1 and AR2 because those SPMs do not contain citations. We made three important assumptions for the calculation. First, we assumed that all references within a specific citation are equally important. Since the citations often refer to multiple chapters, we divided the evidence base of a given statement based on the number of chapters it refers to. For example, given a statement that cites chapters 2 and 3, we calculate that the evidence base statement of that sentence is 50% chapter 2 and 50% chapter 3. Second, we assumed that the citations are exhaustive, which is to say that SPM statements cite all chapters that support their content. Third, we assumed that all statements are equally important, so that all citations are also equally important for the entirety of the SPM. This allowed us to compare the contribution of chapters with different IAM reference shares.

#### Analysis of IAM research

After calculating the contribution of IAM research to the ARs, we sorted the list of researchers from most to least prominent according to their cumulative contribution to each report. We calculated how much each individual researcher contributed to the overall contribution. This allowed us to analyze to what extent any specific individual or group of IAM researchers is dominant. This yielded a list of dominant IAM researchers per report, i.e., the researchers who together (co-)author more than 50% of IAM research in each AR (Table S1). We combined these results with an additional analysis of the gender, affiliated model, and country of the affiliated institution. Further, using the list of dominant IAM researchers (Table S1), we checked to see which (and how many) of them were also AR authors to explore whether AR authorship can explain the (changing) levels of IAM research contribution to WG3 assessments.
