# Supplementary material for: Past peak prominence: The changing role of integrated assessment modeling in the IPCC
Source: iScience. 2024 Oct 19;27(11):111213. doi: 10.1016/j.isci.2024.111213 (PMC11568358; doi:10.1016/j.isci.2024.111213)
Supplement: Document S1. Figures S1–S4 and Tables S1 and S2 [file mmc1.pdf]

iScience, Volume *27*

Supplemental information

Past peak prominence: The changing role of integrated assessment modeling in the IPCC

Ema Gusheva, Stefan Pfenninger, and Johan Lilliestam

Figure S1. Detailed method overview, related to STAR★METHODS. IAM research operationalization (blue), Analysis of IAM research contribution to ARs (green), Analysis of IAM research contribution to SPMs (yellow), and Analysis of IAM research (orange) accompany the same-name sections in STAR★METHODS.

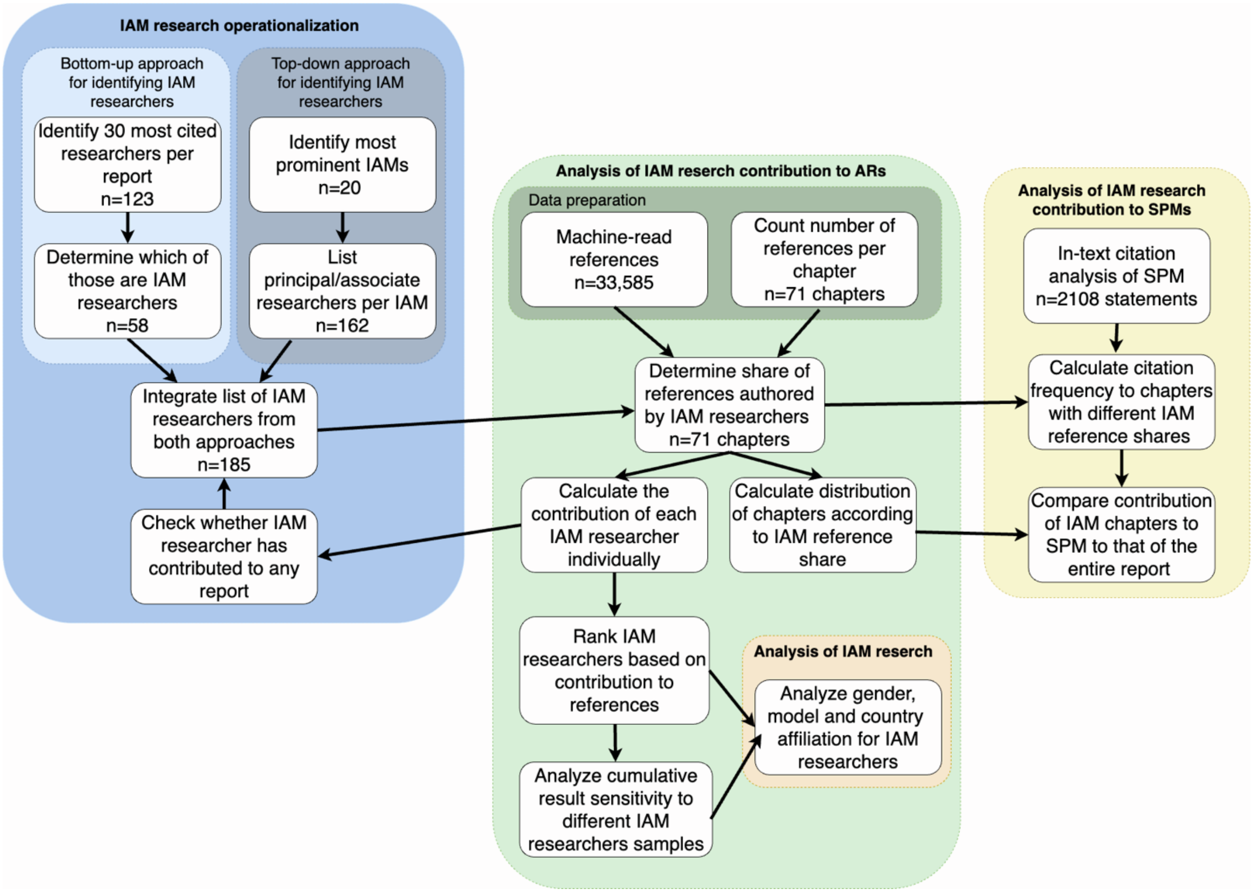

Figure S2. IAM research as share of references over time based on different sample sizes of IAM researchers, related to Discussion and STAR★METHODS. The list of IAM researchers was first sorted based on the size of their contribution to IAM research in the reports. Thus, the line representing 5 researchers represents the top 5 IAM researchers whose contribution was largest.

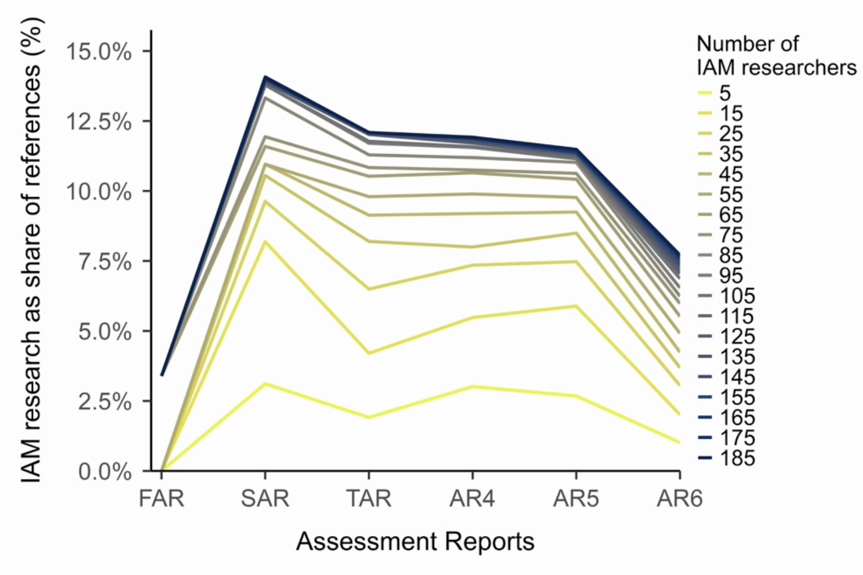

Figure S3. Distribution of IAM research across all report chapters, related to Figure 3a. Figure S3 is an extended version of Figure 3a. Data for AR1 and AR2 is not shown in 3a to keep the comparison to 3b more straightforward.

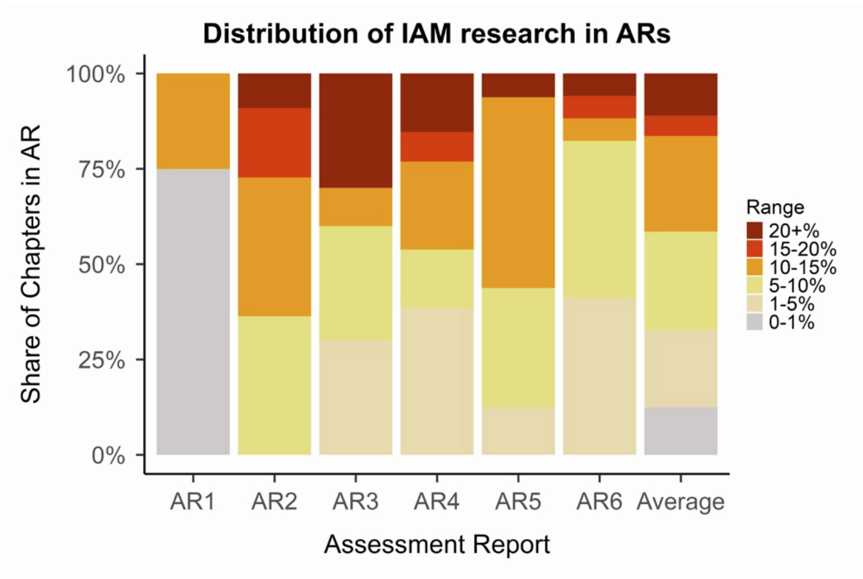

Figure S4: SPM contribution of chapters with a high IAM share, related to Figure 3b. The figure shows how many SPM statements cite a chapter with a high IAM reference share (>20%) unlike results portrayed in Figure 3b, which average out the contribution of different chapters in instances where multiple chapters are cited at once.

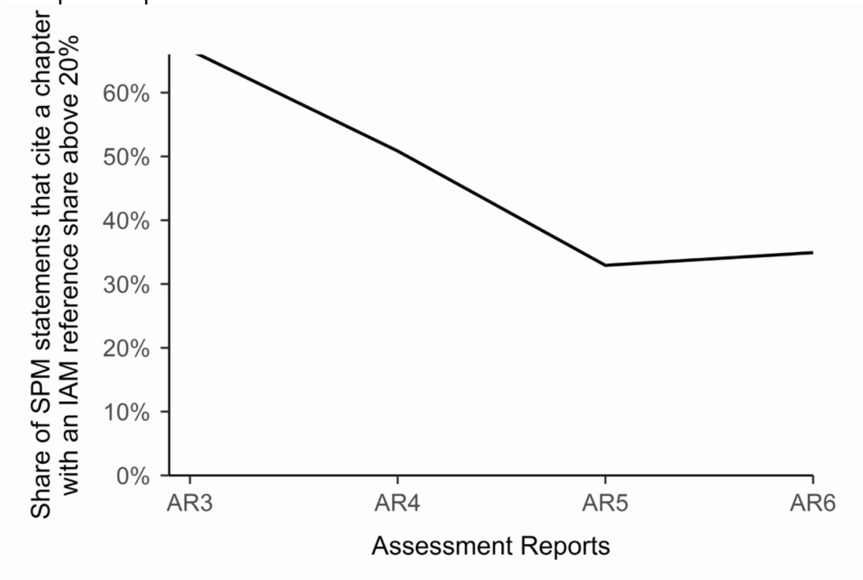

Table S1. AR authorship for dominant IAM researchers who together author >50% of all IAM research in each AR, related to Table 1. The name of the researcher (column 3) is shown alongside the AR they are dominant in (column 1), their (lack) of authorship in that AR (column 4) and the cumulative share of IAM research they author together with all other researchers listed for the same AR (column 2).

Assessment report	Share of total IAM research in AR (co-) authored	Name	AR authorship
AR1	1,00	Gary Yohe	No
AR2	0,5	Michael Grubb	Yes
AR2	0,5	Alan Manne	No
AR2	0,5	Richard Richels	Yes
AR2	0,5	Jae Edmonds	Yes
AR2	0,5	William Nordhaus	No
AR3	0,52	Lynn Price	Yes
AR3	0,52	Richard Richels	Yes
AR3	0,52	Yuzuru Matsuoka	Yes
AR3	0,52	Lawrence Goulder	Yes
AR3	0,52	Carlo Carraro	Yes
AR3	0,52	Jayant Sathaye	Yes
AR3	0,52	Nebojsa Nakicenovic	Yes
AR3	0,52	Michael Grubb	Yes
AR3	0,52	Jae Edmonds	Yes
AR3	0,52	Tsuneyuki Morita	Yes
AR4	0,52	Richard Tol	No
AR4	0,52	John Weyant	Yes
AR4	0,52	André Faaij	Yes
AR4	0,52	Bas Eickhout	Yes
AR4	0,52	Priyardashi Shukla	Yes
AR4	0,52	Jayant Sathaye	Yes
AR4	0,52	Rob Swart	Yes
AR4	0,52	Jae Edmonds	Yes
AR4	0,52	Nebojsa Nakicenovic	Yes
AR4	0,52	Michel den Elzen	Yes
AR4	0,52	Keywan Riahi	Yes
AR4	0,52	Michael Grubb	Yes
AR4	0,52	Detlef van Vuuren	Yes
AR5	0,51	Ottmar Edenhofer	Yes
AR5	0,51	Malte Meinshausen	No
AR5	0,51	Sergey Paltsev	Yes
AR5	0,51	Nico Bauer	No
AR5	0,51	Carlo Carraro	Yes

AR5	0,51	Niklas Höhne	Yes
AR5	0,51	David McCollum	Yes
AR5	0,51	Steve Rose	Yes
AR5	0,51	Shonali Pachauri	No
AR5	0,51	Elmar Kriegler	Yes
AR5	0,51	Katherine Calvin	Yes
AR5	0,51	Jae Edmonds	Yes
AR5	0,51	John Reilly	No
AR5	0,51	Leon Clarke	Yes
AR5	0,51	Massimo Tavoni	No
AR5	0,51	Gunnar Luderer	Yes
AR5	0,51	Volker Krey	Yes
AR5	0,51	Michel den Elzen	Yes
AR5	0,51	André Faaij	Yes
AR5	0,51	Keywan Riahi	Yes
AR5	0,51	Detlef van Vuuren	Yes
AR6	0,51	Christoph Bertram	No
AR6	0,51	Kejun Jiang	Yes
AR6	0,51	David McCollum	Yes
AR6	0,51	Céline Guivarch	No
AR6	0,51	Vassilis Daioglou	Yes
AR6	0,51	Katherine Calvin	Yes
AR6	0,51	Roberto Schaeffer	Yes
AR6	0,51	Priyardashi Shukla	Yes
AR6	0,51	Niklas Höhne	Yes
AR6	0,51	Jia Liu	No
AR6	0,51	Christopher Bataille	Yes
AR6	0,51	Gunnar Luderer	Yes
AR6	0,51	Jean-Charles Hourcade	Yes
AR6	0,51	Michael Grubb	Yes
AR6	0,51	Massimo Tavoni	Yes
AR6	0,51	Shinichiro Fujimori	Yes
AR6	0,51	Charlie Wilson	Yes
AR6	0,51	Michael Jakob	No
AR6	0,51	Elmar Kriegler	Yes
AR6	0,51	Narasimha Rao	Yes
AR6	0,51	Keywan Riahi	Yes
AR6	0,51	Joeri Rogelj	Yes
AR6	0,51	Detlef van Vuuren	Yes

Table S2: Top-down identification of prominant IAMs and IAM researchers.

Model	Information source on modelers	Types of researchers listed	Reason for inclusion in analysis
AIM	Email correspondence	Principal	Listed in AR2 and AR6
WITCH	Email correspondence	Principal	Listed in AR6
IMAGE	Email correspondence	Principal	Listed in AR2 and AR6
GCAM	Email correspondence	Principal	Listed in AR6
MESSAGEixGLOBIOM	[1]	Associate	Listed in AR2 and AR6
MERGE	SAR	Principal	Listed in AR2
COFFEE-TEA	[2–4]	Associate	Listed in AR6
EPPA	[5–7]	Associate	Common in MIPs
REMIND-MAgPIE	[8–11]	Associate	Listed in AR6
DNE21	[12]	Associate	Common in MIPs
DIAM	SAR	Principal	Listed in AR2
IPAC	[13–15]	Associate	Common in MIPs
FUND	SAR	Principal	Listed in AR2
CETA	SAR	Principal	Listed in AR2
DICE	SAR	Principal	Listed in AR2
IMACLIM-R	Email correspondence	Principal	Listed in AR6
TIAM	[16–18]	Associate	Listed in AR6
POLES	[19,20]	Associate	Listed in AR6
GEM-E3	[21]	Associate	Listed in AR6
PRIMES	[22]	Associate	Common in MIPs

Supplemental references

1. Huppmann, D. *et al.* The MESSAGEix Integrated Assessment Model and the ix modeling platform (ixmp): An open framework for integrated and cross-cutting analysis of energy, climate, the environment, and sustainable development. *Environmental Modelling & Software* **112**, 143–156 (2019).
2. Cunha, B. S. L., Garaffa, R. & Gurgel, Â. C. *TEA Model Documentation*. http://bibliotecadigital.fgv.br:80/dspace/handle/10438/28756 (2020).
3. IAMC. COFFEE – COmputable Framework For Energy and the Environment model – iamconsortium. https://www.iamconsortium.org/resources/model-resources/computable-framework-for-energy-and-the-environment-model-coffee/.
4. Rua Rodriguez Rochedo, P. Development of a global integrated energy model to evaluate the brazilian role in climate change mitigation scenarios. (2016).
5. Babiker, M. H. M. *et al.* The MIT Emissions Prediction and Policy Analysis (EPPA) model : revisions, sensitivities, and comparisons of results. *MIT EPPA model* (2001).
6. Babiker, M. M. H., Gurgel, A. C., Paltsev, S. & Reilly, J. M. *A Forward Looking Version of the MIT Emissions Prediction and Policy Analysis (EPPA) Model*. https://dspace.mit.edu/handle/1721.1/44618 (2008).
7. Paltsev, S. *et al. The MIT Emissions Prediction and Policy Analysis (EPPA) Model: Version 4*. https://dspace.mit.edu/handle/1721.1/29790 (2005).
8. Baumstark, L. *et al.* REMIND2.1: transformation and innovation dynamics of the energy-economic system within climate and sustainability limits. *Geoscientific Model Development* **14**, 6571–6603 (2021).
9. Dietrich, J. P. *et al.* MAgPIE 4 – a modular open-source framework for modeling global land systems. *Geoscientific Model Development* **12**, 1299–1317 (2019).
10. Lotze-Campen, H. *et al.* Global food demand, productivity growth, and the scarcity of land and water resources: a spatially explicit mathematical programming approach. *Agricultural Economics* **39**, 325–338 (2008).
11. Luderer, G. *et al.* Description of the REMIND Model (Version 1.6). SSRN Scholarly Paper at https://doi.org/10.2139/ssrn.2697070 (2015).
12. Akimoto, K., Tomoda, T., Fujii, Y. & Yamaji, K. Assessment of global warming mitigation options with integrated assessment model DNE21. *Energy Economics* **26**, 635–653 (2004).
13. Jiang, K., Zhuang, X., Miao, R. & He, C. China's role in attaining the global 2°C target. *Climate Policy* **13**, 55–69 (2013).
14. Jiang, K. *et al.* Energy transition, CO2 mitigation, and air pollutant emission reduction: scenario analysis from IPAC model. *Nat Hazards* **99**, 1277–1293 (2019).
15. Jiang, K. & Hu, X. Energy demand and emissions in 2030 in China: scenarios and policy options. *Environ Econ Policy Stud* **7**, 233–250 (2006).
16. G. Anandarajah, S. Pye, W. Usher, F. Kesicki, C. Mcglade, TIAM-UCL Global model documentation, UCL, 2011.
17. Loulou, R. & Labriet, M. ETSAP-TIAM: the TIMES integrated assessment model Part I: Model structure. *CMS* **5**, 7–40 (2008).
18. van der Zwaan, B., Kober, T., Longa, F. D., van der Laan, A. & Jan Kramer, G. An integrated assessment of pathways for low-carbon development in Africa. *Energy Policy* **117**, 387–395 (2018).
19. Criqui, P. International markets and energy prices: the POLES model. in *Models for Energy Policy* (Routledge, 1995).
20. Despres, J. *et al.* POLES-JRC model documentation - Updated for 2018. *JRC Research Reports* (2018).
21. Capros, P. *et al.* GEM-E3 Model Documentation. *JRC Publications Repository* https://publications.jrc.ec.europa.eu/repository/handle/JRC83177 (2013) doi:10.2788/47872.
22. Capros, P., Tasios, N., De Vita, A., Mantzos, L. & Paroussos, L. Model-based analysis of decarbonising the EU economy in the time horizon to 2050. *Energy Strategy Reviews* **1**, 76–84 (2012).
